# Intimate partner violence (IPV) prevention using a cross-sectoral couple-based intervention: results from a cluster randomised control trial in Ibadan, Nigeria

**DOI:** 10.1136/bmjgh-2021-007192

**Published:** 2022-02-09

**Authors:** Neetu A John, Ayodeji Adebayo, Natalie A Boychuk, Funmilola OlaOlorun

**Affiliations:** 1 Heilbrunn Department of Population and Family Health, Columbia University Mailman School of Public Health, New York, New York, USA; 2 Department of Community Medicine, College of Medicine, University of Ibadan, Ibadan, Oyo, Nigeria

**Keywords:** public health, cluster randomized trial, KAP survey, qualitative study

## Abstract

**Introduction:**

Intimate partner violence (IPV) is the most common form of violence women experience globally. Economic empowerment interventions have been implemented across countries to prevent and address IPV, with mixed results. A sociological ‘male-backlash’ model suggests that addressing unequal gender norms is crucial to reduce IPV. This study evaluates the impact of a multipronged intervention among heterosexual couples in urban and periurban Ibadan that aimed at reducing IPV by increasing financial and reproductive literacy, fostering gender equality and improving relationship quality.

**Methods:**

A four-arm mixed-methods cluster randomised control trial was employed. Baseline data and end line data six months postintervention were collected to estimate changes in key outcomes. In-depth interviews were conducted with 15 couples 2 years postintervention to explore the drivers of changes in outcomes. Difference-in-differences regression models were estimated to compare changes in IPV levels across the three intervention arms and control arm, and thematic analysis was conducted to understand drivers of change in IPV outcomes.

**Results:**

Physical IPV decreased significantly in the gender socialisation (GS) (β: −4.63 (SE: 2.12)) and GS and financial literacy (β: −4.61 (SE: 2.02)) groups as compared with the control group. Changes in emotional and sexual IPV were marginally significant or insignificant, respectively, suggesting that the intervention did not have an impact on non-physical forms of IPV. In the in-depth interviews, couples reported improved communication and trust, enhanced conflict management skills, and increased mutual respect as a result of participation across intervention arms, which may have facilitated the reduction of violence in their relationships.

**Conclusion:**

This study highlights the potential utility of gender transformative interventions for improving physical IPV outcomes. Future research should seek to understand the mechanisms that influence sexual and emotional IPV as their aetiology may be different from physical violence.

**Trail registration number:**

The study protocol was registered at ClinicalTrials.gov (ID: NCT03888495).

**Key questions**

**What is already known?**

- Women’s economic empowerment has been used to address intimate partner violence (IPV), with mixed results.

**What are the new findings?**

- Fostering women’s empowerment within the household by targeting couples and addressing gender norms and strengthening relationship can reduce IPV, particularly physical IPV.
- In-depth interviews found that the intervention improved overall relationship quality by increasing mutual respect, improving communication and building conflict resolution skills within partnerships. These impacts were sustained over time.

**What do the new findings imply?**

- Structural interventions may not be sufficient in the short term to shift the underlying gender-power dynamic and oppressive gender norms that promote IPV.
- The gender socialisation component of the intervention, which focused on critical reflection, skills building and relationship strengthening to foster egalitarian spousal relationships, may have been a critical mechanism for reducing IPV in this population.

## Introduction

Intimate partner violence (IPV) is the most common form of violence that women experience globally. One in three women experience IPV at some point in their life, based on cross-national estimates across 80 countries.^1^ The levels of IPV are much higher for low-resource settings such as South Asia and sub-Saharan Africa.^1^ In the African, Eastern Mediterranean and South-East Asian WHO regions, the prevalence of IPV is almost 40%.^2^ Several studies, including meta-analyses, have demonstrated the direct and indirect ways exposure to different forms of IPV harm a woman’s mental, physical and reproductive health; for example, IPV leads to an increased risk of adverse birth outcomes, unintended pregnancies and abortions.^3–5^ Beyond health, IPV can have socioeconomic consequence such as lost wages, social isolation and decreased participation in regular activities.^6^ Children living in households with elevated levels of IPV are also susceptible to adverse health and development outcomes.^7^ Exposure to IPV can increase risk of infant and child mortality, and lead to impaired growth and development, with long-term health, behavioural and social consequences.^8–10^

Given the magnitude of IPV and its effects on health and well-being of women and their families, reducing IPV is a significant global health priority and a key indicator for Sustainable Development Goal 5: achieving gender equality and empowering all women.^1^ Initial approaches for IPV reduction drew on economic models of household bargaining and assumed that women’s participation in paid work and ownership of assets would increase their bargaining power within households, and reduce risk of experiencing IPV.^11–13^ These approaches were subsequently criticised for ignoring powerful contextual factors, such as prevailing gender norms, which condone and perpetuate gender inequalities.^14–16^ Drawing on sociological ‘male-backlash’ models, critics argued that by entering wage labour, women are essentially challenging existing socially prescribed male breadwinner roles, which may increase IPV experienced as men try to shift the balance of power towards themselves by inflicting violence on women.^17–19^ These models are particularly relevant in settings where divorce is not normative and ending a relationship comes with significant stigma, making exiting a relationship unviable for many women.^20^ ^21^

Mirroring these theories, evaluations on the effects of standalone economic interventions on IPV have returned mixed results. A 2009 systematic review found that the relationship between economic empowerment and IPV is highly context specific, with additional income leading to increases in violence in some settings.^22^ A recent systematic review also reported mixed results on the relationship between IPV and standalone cash transfer and micro-credit programmes. They found that participation in these programmes can lead to increases in physical violence, particularly when the woman has a higher level of education, higher decision-making power, resides in an ‘urban village,’ and receives a larger amount of funds.^18^ A randomised roll-out of an unconditional cash transfer programme in Ecuador to mothers investigated how an exogenous increase in women’s income affects IPV.^23^ The authors found that the effect of a cash transfer depended on a woman’s level of education, as well as her education, relative to her husband. While for women with greater than primary school education, cash transfers decreased emotional IPV, for women with less that primary school education, the effect of the cash transfer varied relative to her education and that of her husband. Among these women, the cash transfer increased emotional IPV if the women’s education was equal to or more than her husband. Other evaluations of microcredit programmes have also indicated that these programmes are unlikely to increase women’s bargaining power as women are not able to control their resources and often give their loans to their husbands.^24–26^

Despite the conflicting evidence, stand-alone women’s economic empowerment interventions continue to be touted as magic bullets to foster gender equality. Clearly, while the focus on women’s economic empowerment is critical, simultaneous efforts need to be made to address the underlying gender norms that perpetuate gender inequality, at least in the short-term, particularly, in contexts where women have limited freedom to dissolve their marriages, leave their partners, and take their families and property with them. Programmes, however, rarely take a multivalent approach, one that simultaneously addresses the structural and normative barriers that frustrate women’s empowerment and increase the risk of violence.^24^ Recently, a few randomised studies from Africa combined economic empowerment approaches with gender components and have shown promising effects in reducing IPV.^27–29^ Nevertheless, many of these programmes continue to be narrow in scope as they tend to focus on the woman alone or target men tangentially, or their curriculum does not build critical relationship skills that may foster shared decision-making and an egalitarian relationship that is free from violence and coercive behaviours. The long-term impacts of these interventions on IPV reduction are also yet to be studied.

Given the limitations in existing programmes, the study team implemented a cluster-randomised control trial to test the impact of a multisectoral programme seeking to promote women’s empowerment within the household. The programme targeted key domains of women’s disempowerment within the household such as unequal spousal relationships and limited decision-making ability in the financial and reproductive arena. These domains were also targeted because of their potential for synergistic impacts on fostering women’s empowerment. While economic empowerment has been theorised to increase a woman’s bargaining power, reproductive empowerment is hypothesised to reinforce these benefits by giving women the time and space to take control of their life and relationships.^30–32^ However, these benefits are unlikely to accrue without an enabling household environment, where women feel supported and are able to exercise their agency and choice^33^ Therefore, the gender socialisation (GS) intervention, which addresses harmful gender norms and inculcates egalitarian spousal relationships was the primary intervention and all intervention arms received this intervention. The GS intervention was layered with components on financial literacy education and contraceptive counselling in the subsequent arms to bolster women’s empowerment.

We assessed the impact of the intervention on numerous women’s empowerment measures,^34^ including IPV. In this paper, we discuss the impact of the programme on IPV. The study was implemented among young couples, where the wife was between 18 and 35 years old in urban and periurban Ibadan. The study team collected baseline and endline survey data. The endline survey data were collected 6 months after the intervention ended. Additionally, the team conducted in-depth interviews with couples across the different intervention arms 2 years after conclusion of the programme to explore the programme’s sustained influence over time and the drivers by which the programme impacted IPV experiences within couples.

## Methods

### Study population

Women aged 18–35 years and their coresiding spouses or long-term partners living in selected communities in Ibadan, Nigeria were eligible for recruitment into the study. Participants were recruited from two urban (Ibadan North, Ibadan Southwest) and two peri urban (Akinyele, Oluyole) local government areas (LGAs) between September 2017 and July 2018.

### Study design

The study was designed as a four-arm cluster randomised control trial. Cluster randomisation rather than individual randomisation was considered appropriate for this study as communities often tend to cluster around people with similar sociodemographic characteristics, making it hard to apply the principle of independence for socially determined behaviour. In arm 1, couples received GS training as well as relationship education and skills building (GS arm). In arm 2, couples received financial literacy training, which included sessions on household financial planning and budgeting, in addition to GS training (GS and financial literacy, GSFL arm). In arm 3, couples received all three interventions: GS training, financial literacy education and contraceptive counselling with vouchers for couples from the poorest quintile to secure a method of their choice (ALL arm). Arm 4 served as the control group. Group assignment was not masked for participants or members of the study team, given the nature of the study.

A mixed-methods study design was used to collect data. Baseline and endline survey data were collected from the intervention and the control arms to estimate any change in key outcomes, including IPV measures. The endline data were collected 6 months after conclusion of the intervention. Additionally, 2 years postintervention, in-depth interviews were conducted among 15 couples (spouses interviewed separately) in the intervention arms to examine the mechanisms by which the programme may have impacted IPV prevalence.

### Sample size determination

The minimum sample of study participants needed in each arm of the study was calculated for key outcome measures including IPV using raw data available for Oyo state from the Nigeria Demographic and Health survey conducted in 2013, assuming a fixed number of clusters (n=12 per arm).^35 36^ We assumed the intervention would improve key outcomes by 15 percentage points. Assuming 80 percent power, a two-sided type 1 error of 5%, the largest sample size (225 participants per arm) was needed to detect changes in household decision-making based on the proportion (0.275) of married/cohabiting women aged 18–35 years, who participated in these decisions. An intraclass correlation coefficient of 0.015 was assumed in the calculations, as well as a fixed number of clusters of 12 per arm (48 in all). Adjustments for 20% lost to follow-up rates give a total of 282 couples per study arm. Calculations based on the above assumptions gave a minimum sample size of 1128 couples (~24 couples/per cluster).

### Randomisation

Study couples were selected through a three-stage block randomisation process with stratification conducted by an independent statistician.^37^ The 11 LGAs of Ibadan, 5 urban and 6 periurban, were divided into two equal halves using geographical boundaries. An urban and peri urban LGA were randomly selected from each half using a random number generator, giving a total of four LGAs, which served as the strata. A fixed number of clusters (n=12) were then randomly selected in each of the four selected LGAs. To ensure geographically distinct clusters within each LGA, alternate distinct localities were selected using a map of the LGA in a serpentine fashion, following a random start. A cluster composed of one randomly selected index enumeration area, and its adjoining enumeration area with a higher numerical code.^35^ Where a sufficient number of couples (~24 couples per cluster) could not be recruited from these two enumeration areas, the cluster was expanded to geographically adjacent enumeration areas. Household-listing was conducted in the selected EA clusters to enable identification and recruitment of eligible couples per study arm to participate in the study. In general, where more than 26 eligible couples were listed in each cluster, systematic random sampling, following a random start was used to select couples. Couple selection only took place once, at the beginning of the study.

### Study interventions

The intervention consisted of a package of three interventions*—*GS training, financial literacy education and contraceptive counselling. The first study arm (GS arm) received four sessions on GS. These sessions were focused on building knowledge, awareness, critical consciousness around power, care work and gender inequalities. Additionally, the sessions sought to build skills in egalitarian decision-making, conflict management, negotiation, and communication. In arm 2 (GSFL), in addition to GS training, the participants received three sessions on financial literacy and household budget management. Participants were introduced to key financial terms as well as trained on financial planning, decision making and household budgeting. Arm 3 (ALL) received all three interventions. In addition to the GS and GSFL training, they received an additional session on contraceptive counselling. The content of the family planning session was the same as the counselling offered by nurses in family planning clinics in Ibadan, Nigeria, where the study took place.

The intervention was implemented over a 6-week period from 28 July 2018 to 8 September 2018, starting almost immediately after baseline interviews were completed. The sessions were offered weekly at the cluster level, with approximately 24–26 couples in attendance. Each session was 2 hours long. The GSFL sessions were led by experienced and well-trained facilitators, and included individual and group activities, as well as role play. The couple contraceptive counselling session was led by a family planning nurse trained by the Nigerian Urban Reproductive Health Initiative using the Balanced Counselling Strategy.

The training materials for GSFL training were contextually adapted from relevant existing materials obtained through consultation with experts in the field as well as search of the literature. While some sessions were intentionally gender-disaggregated, others brought the couple together, particularly sessions that focused on skill-building.

### Study retention

The study lost 14% of the couple sample at endline follow-up. At baseline, 1236 couples were recruited into the study, with 307 in the GS arm, 299 in the GSFL arm, 299 in the ALL arm and 331 in the Control arm. At endline, only 1064 couples could be reached, with 261 in the GS arm, 256 in the GSFL arm and 259 in the ALL arm, and 288 in the Control arm. At endline, we interviewed the available partner after three attempts of tracking the partner. This led to 16 additional interviews with individual women (GS=3, GSFL=6, ALL=1, control=6), and 16 interviews with individual men (GS=5, GSFL=3, ALL=5, control=3). Couples lost to follow-up had either moved out of the city or were unavailable at their residence after repeated visits and were not reachable by phone. The study’s consort flow diagram can be found in a previous publication.^34^

### Study procedure

#### Surveys

Baseline interviews, using paper-based structured questionnaires took place between 15 September 2017 and 10 July 2018, prior to randomisation of clusters. Couples were interviewed simultaneously either in a public space, such as a public hall or a hotel meeting room, or in their homes, based on their preference. All efforts were made to ensure auditory and visual privacy, including using strategies such as interviewing the couple at the same time to structurally ensure the partners do not overhear each other’s interviews. Women and their partners were interviewed separately by gender-matched field staff who worked together in pairs. The female interviewer interviewed the wife, while the male interviewer interviewed the husband. If one member of the couple was not available, the interview was rescheduled for a time when both partners would be available. The interviews were conducted after both partners consented to participate. Endline interviews were conducted 6 months postintervention and followed the same protocol followed at baseline. Efforts were made by interviewers to reach all couples who had been interviewed at baseline, even if they had moved outside of the community.

#### In-depth interviews

In-depth interviews were conducted with 15 couples 2 years after the intervention concluded. Five couples each were randomly selected from each of the intervention arms. The spouses were interviewed separately by trained gender-matched interviewers. The interviews were conducted after both partners provided consent. The interviews were held either at the respondent’s home or outside based on their preference. Every effort was made to ensure auditory and physical privacy. The goal of the interviews was to investigate the mechanisms by which the programme may have contributed towards reductions in IPV. The duration of interview ranged from 45 to 60 minutes. The interviews were audiorecorded with permission from the respondent.

### Measures

#### Intimate partner violence

The IPV measures were adapted from the WHO’s Multi-Country Study on Women’s Health and Domestic Violence Against Women.^38^ The survey asked female respondents to report different forms of IPV (physical, emotional and sexual) before and since the conclusion of the intervention. Physical IPV was measured with a series of questions that asked a woman if her husband/partner had done the following: slapped or had something thrown at her that could hurt her; pushed or shoved her; hit her with their fist or something else that could hurt her; kicked, dragged or beaten or choked or burnt her on purpose; threatened to use or actually used a gun, knife or other weapon against her. For sexual IPV, they were asked if their husband/partner had perpetrated following acts: physically forced them to have sexual intercourse when she did not want to; had sexual intercourse when she did not want to because she was afraid of what her partner might do if she refuses; was forced to do anything sexual that she did not want to do. Finally, for emotional IPV, the women were asked if their husband or partner had: insulted or made them feel bad about themselves; belittled or humiliated them in front of other people; scared or intimidate her on purpose, for example, by the way he looked at her, by yelling or smashing things; threatened to hurt someone she cared about.

For every indicator of physical, sexual and emotional IPV, the woman was asked if she had experienced the event once, a few or many times. The woman was provided information on the content of survey prior to the interview and gave her consent. She was reminded of her rights to discontinue and not answer any question prior to the section on IPV.

The IPV measures were constructed with principal component analysis as separate indices that captured the intensity of different forms of violence experienced and took a value between 0 and 100, where a higher value indicates more violence. This approach was preferred over the conventional method of dichotomising and coding physical and sexual IPV together as research suggests that dichotomisation misses important nuances, especially in contexts such as Nigeria, where IPV is endemic.^39^ The results also broadly remain the same if we use binary IPV measures.

#### Intervention groups

Three dummy variables differentiated between intervention and control group. They were labelled GS Arm, GSFL Arm, and ALL Arm for the group that received the family planning intervention in addition to the first two interventions.

#### Background variables

Key socioeconomic and demographic variables were used to adjust for systematic differences between the groups as could be expected to emerge in clustered samples. Age was measured continuously in years. Religion was constructed as a binary variable to differentiate Christians from Muslims. Ethnicity distinguished Yorubas from other groups. Education was measured as a categorical variable to separate those with no education, primary and secondary or higher education. Paid cash distinguished women who received payment for their work from women who did not receive payments or were not working. Polygamous marriage was measured as a dummy variable. Number of children represents average number of living children.

### Analyses

#### Quantitative

We assessed for systematic differences in key demographic characteristics and key outcomes between the final intervention sample and the sample lost to follow-up. The impact of the programme was assessed with difference-in-differences (DID) regression models to assess changes over time in IPV prevalence in the intervention arms compared with the control arm. DID identifies programme impact as the difference in the change observed in an outcome between participants and non-participants over the interval of the programme. A key assumption is that in the absence of the treatment, the difference between the intervention and control group would be constant over time, or the ‘trend’ of change would be the same in the groups. DID was implemented as an interaction term between the time and treatment group dummy variables in a regression model and can be specified as indicated below. The models were adjusted for clustering as well as covariates that were unbalanced between the intervention and control arms. All analyses were conducted in Stata V.15.

Y=β0+β1∗[Time]+β∗[intervention]+β3∗[Time∗Intervention]+β4∗[Covariates]+ϵ

#### Qualitative analysis

Qualitative analysis was conducted using a deductive thematic analysis. All analyses were conducted using NVivo V.12. First, major themes were identified from the transcripts. Next, a codebook was developed and refined through an iterative process. Codes and subcodes were given clear definitions and descriptions of when to use and when not to use to avoid overlap between codes. Super codes and families were used to facilitate the categorisation of codes. Two research assistants with qualitative research experience were trained by the lead qualitative researcher on how to use the codebook. They coded the first three transcripts with the lead researcher. Thereafter, they coded the transcripts independently and met to compare their analysis and resolve any discrepancies, including disagreements in the interpretation of a code, until a consensus was reached. Finally, codes were organised around emergent themes and categories using an inductive approach.

## Results

### Quantitative findings

The final analytical sample consisted of 1080 women, with 264 in the GS arm, 262 in the GSFL arm, 260 in the ALL arm and 294 in the control arm. The sample had less than 1% missing data across variables, and median values were imputed to address them. The loss to follow-up sample and the full sample were comparable but for a few demographic characteristics. The women in the lost to follow-up sample were more likely to be less educated and belong to a non-Yoruba ethnic group. There were no differences in levels of IPV between the two samples.

Sociodemographic characteristics of the participants is shown in table 1. The groups were balanced in key demographic characteristics such as age, number of children and levels of polygamy. However, the GS (60.61 %) and GSFL (61.07 %) arms had significantly higher proportion of Christians as compared with the control group (53.06 %). The GSFL arm had a slightly lower proportion of Yoruba (75.57%), and a more diverse ethnic profile as compared with the control group (82.31%). Lastly, women in the GS arm were more likely to have higher education as compared with the control group. Our DID models adjusted for these differences between samples.

**Table 1:** Demographic characteristics of the sample at baseline

Variable	Control (n=294)	GS (n=264)	GSFL (n=262)	ALL (n=260)
Age in years	28.63 (4.15)	29.37 (4.19)	28.98 (4.33)	28.39 (4.32)
Religion				
Christians	53.06	**60.61**	**61.07**	56.15
Muslims	46.94	**39.39**	**38.93**	43.85
Ethnicity				
Yoruba	82.31	80.3	**75.57**	80.77
Others	17.69	19.7	**24.43**	19.23
Schooling				
No schooling	7.14	**2.65**	10.31	7.69
Primary	15.99	**14.39**	15.65	19.23
Secondary	55.78	**48.86**	46.18	52.31
Higher	21.09	**34.09**	27.86	20.77
Paid employment	91.94	92.28	90.46	86.34
Polygamy	8.84	11.72	12.21	10.79
No of children	1.60 (1.24)	1.55 (1.35)	1.39 (1.18)	1.51 (1.33)

Values are percentages or means with SD in parentheses. Significant differences between intervention and control arm are bolded.

ALL, Arm with all three interventions; GS, gender socialisation; GSFL, GS and financial literacy.

Table 2 provides mean IPV scores (range: 0–100) of women at baseline and endline. Among forms of violence, women experienced emotional IPV the most, followed by physical and sexual IPV. Mean emotional IPV scores were lowest for the control group and highest in the GSFL group.

**Table 2:** Mean baseline and endline intimate partner violence (IPV) scores (range 0–100) by intervention arms

Variables	Baseline (range: 0–100)				Endline (range: 0–100)			
	Control (n=294)	GS (n=264)	GSFL (n=262)	ALL (n=260)	Control (n=294)	GS (n=264)	GSFL (n=262)	ALL (n=260)
Emotional IPV	14.57 (21.17)	16.33 (23.10)	22.02 (27.31)	17.54 (23.10)	13.65 (22.85)	13.07 (21.79)	15.14 (24.12)	11.73 (18.80)
Physical IPV	4.92 (14.60)	7.21 (19.56)	8.00 (20.64)	5.32 (13.24)	5.63 (16.43)	3.28 (10.40)	4.08 (15.30)	3.13 (11.63)
Sexual IPV	6.89 (17.96)	5.25 (14.19)	8.47 (20.06)	9.00 (19.65)	6.43 (15.75)	8.53 (17.97)	8.28 (19.05)	6.21 (15.46)

ALL, Arm with all three interventions; GS, gender socialisation; GSFL, GS and financial literacy.

Results from the DID regression models can be found in table 3. Physical IPV reduced significantly by around five points in the GS and GSFL arms as compared with the control group, with marginal reductions in the group that received all three interventions. The coefficients for emotional IPV trended in the right direction indicating reductions in emotional IPV in the intervention arms as compared with the control arm but were only marginally significant. The coefficients for sexual IPV increased in the arm that received all three interventions and decreased in the other arms as compared with the control arm but none of the coefficients reached statistical significance.

**Table 3:** Difference-in-difference estimates of programme impact on types of intimate partner violence (IPV)

Intervention arms	Physical IPV	Emotional IPV	Sexual IPV
	β (SE)	β (SE)	β (SE)
**(Ref: control)**			
GS	−4.63 (2.12)**	−2.36 (2.20)	3.74 (3.50)
GSFL	−4.61 (2.02)**	−5.96 (2.9)*	0.28 (3.41)
All	−2.99 (1.69)*	−4.90 (2.77)*	−2.49 (2.15)

Note: Numbers in parentheses are robust 95% CIs.

Models were adjusted for clustering and unbalanced covariates.

*P<0.05, **p<0.01.

GS, gender socialisation; GSFL, GS and financial literacy.

### Qualitative findings

In-depth interviews with participants 2 years postintervention highlighted continued ways in which the intervention across the different arms supported couples to improve their overall relationship quality, enhance communication and reduce conflict, which in turn led to a perceived reduction in IPV.

#### Mutual respect

Programme participants across arms reported that the intervention significantly increased respect between partners, which in turn improved overall relationship quality. The underlying philosophy of the intervention centered around men and women’s equal partnership in a marriage; participants reported that this philosophy helped them reconceptualise their relationship. Increased respect between partners and increased relationship quality more broadly, as participants suggested that their partners became happier and more fulfilled in their relationship:

Like the very first day the program started, the way they approached us as husband and wife, in fact, it was really lovely……., they let us [made us] know that we were meant for each other, that we have equal rights, that we can do things together, that we, that we can achieve at the future; how to come together, how to do things together, then how to share things. So, that’s what I can remember. (Female; GSFL arm)

#### Improved communication/trust

Many respondents across arms noted that the quality of their communication with their partner improved as a result of the intervention. Respondents reported joyful and happy experiences with their partner and identified them as their ‘best friend’ or ‘sole confidant’:

In recent times ahaa!! We do like emm [act like]… sister and brother so we do talk, play, gist, explain the things that happened in our place of work so we pray and then so it has really changed…. …… he has really changed, (Female; ALL arm)

Improvements in communication improved equality of decision-making within couples, as some participants reported that their husbands were more transparent and open to discussions with their partner. Women reported feeling as though their husband was being completely honest and transparent with them.

I’ve said before that our communication now is different unlike before when it was about I’m the man, I’ll let you know only what you need to know, but now (hisses) I can say I know almost everything. So, now is better than before, (Female; GS arm)

#### Conflict management

The benefits of the intervention for communication between partners also helped participants prevent or de-escalate conflict when disagreements arise. Participants noted that before the programme, communication was generally poor, with conflicts often resolved through shouting or phone conversations. As a result, participants often harboured negative feelings towards their partner, which had negative effects on the overall quality of the relationship. Participants across intervention arms suggested that the programme improved their ability to manage anger and frustration.

well, like I said the (the) training helped before I use to be temperamental, hot-tempered but, I will never lay my hands on my wife. But I'll rather keep malice or verbal stuff but I will never hit her. That’s just the only thing that I do. But now, I'm more matured…. (Male; GS arm)

Participants in all three intervention arms reported that taking part in the programme improved their communication and ability to deal with disagreements. Some couples noted that disagreements are now resolved through in-person conversations that value equal input of both partners. Some participants noted that they previously resolved disputes through telephone conversations or delayed speaking with their partner about conflicts, but that they now prioritise speaking in person and resolving disagreements before going to sleep to ensure that issues are resolved.

before we quarrel, argue, but now instead of quarrelling, we will rather sit down and talk about things to do in future, (Female; GSFL arm)

## Discussion

Overall, the quantitative results indicate that the programme was successful in reducing physical IPV, with significant reductions in GS and GSFL arm as compared with the control arm. For emotional IPV, although trends in coefficients suggested a decline in all three intervention arms, these effects were only marginally significant in the GSFL arm and the arm with all three interventions (ALL arm). There were no significant reductions in sexual IPV across the three arms. Additionally, the in-depth interviews suggest that the GS component of the intervention across the different arms had a sustained positive influence in shifting traditional gender norms and improving spousal relationships. Both female and male respondents across the different intervention arms discussed how participation in the programme fostered egalitarianism, mutual respect and improved spousal relationship quality by reducing conflict, enhancing communication and building trust. Since no significant incremental reductions in violence were observed in layering the GS intervention with financial literacy and contraceptive counselling components, and this together with findings from the in-depth interviews; the GS intervention with its focus on tackling underlying gender norms and unequal spousal relationships appears to be the critical mechanism that facilitated changes in household dynamics and violence reduction.

Our study adds to a growing body of studies demonstrating that structural interventions may not be sufficient to shift oppressive gender norms and power dynamics that promote IPV, at least in the short term, and these norms and dynamics need to be tackled head-on. This has motivated an interest in gender-transformative interventions to shift household dynamics and reshape spousal relationships.^40 41^ Recently, a few randomised control trials have demonstrated the effectiveness of targeting couples to reduce IPV. The Bandebereho and Indashyikirwa trials in Rwanda, involving group sessions with couples have resulted in significant reductions in physical and sexual IPV.^29 42^ A randomised controlled trial of elderly couples in Shirz, Iran involving an ‘emotion-focused psychoeducational intervention’ similarly found that emotional abuse decreased significantly among couples who participated in the intervention.^43^ Despite concerns that conjoint interventions for couples experiencing IPV may increase harm or retaliation against survivors, there is evidence to suggest that in some circumstances, couple-centred interventions can address the underlying relationship dynamics exacerbating violent behaviour.^44^ These successes as well as the results from our study indicate that a couple-centred approach can be an effective way to reduce IPV. While we do not have cost-effectiveness data from our study, recent estimates from other programmes indicate that a couple’s approach may be a cost-effective strategy, and that these costs may further go down as programmes are scaled up.^44^

Our study was unique in its focus on enhancing women’s empowerment within the household by targeting three critical domains of disempowerment: spousal relationship, financial decision-making and reproductive power. Although the intervention did not directly discuss IPV, its emphasis on shifting couple relationship dynamics by targeting traditional gender norms and providing relationship education and skills-building was effective in reducing physical IPV, and these positive changes appear to have sustained over time. These results highlight how gender power differentials that perpetuate violence and other harmful practices are amenable to sustainable change even with a brief targeted intervention in some settings. However, our programme marginally changed emotional IPV and did not impact sexual IPV suggesting the aetiology of different types of violence may be different, which future research should explore. It was rigorously evaluated using a cluster randomised control trial and reached over 1000 couples and we conducted a qualitative follow-on study to explore if changes were sustained over time. Despite its strengths, there are several limitations that require discussion. We were unable to mask group assignment from participants. The key measures were self-reported, and therefore, prone to social desirability bias. Our findings are not generalisable beyond the population of urban Ibadan and similar settings.

## Data Availability

Data are available on reasonable request.
